# Giant Ovarian Mucinous Tumor Managed by Transvaginal Drainage of Tumor Contents Followed by Laparoscopic Resection: A Case Report

**DOI:** 10.7759/cureus.111960

**Published:** 2026-07-02

**Authors:** Sho Mori, Mikiya Kato, Takeru Murai, Yasuyuki Kishigami, Hidenori Oguchi

**Affiliations:** 1 Obstetrics and Gynecology, TOYOTA Memorial Hospital, Toyota, JPN

**Keywords:** giant ovarian tumor, laparoscopic surgery, minimally invasive surgery, ovarian mucinous tumor, transvaginal drainage

## Abstract

Laparoscopic surgery is widely preferred for benign ovarian tumors because it is associated with less postoperative pain, fewer complications, and shorter recovery time compared with laparotomy. However, laparoscopic surgery for giant cystic ovarian tumors occupying the entire abdominal cavity poses substantial challenges, including difficult trocar insertion and the risk of intraperitoneal spillage, particularly when the cyst contains highly viscous mucinous material that is difficult to aspirate with standard suction devices. We report a case of a 72-year-old woman with a 37.7 × 32.0 × 15.5 cm multilocular ovarian tumor extending from the pouch of Douglas to the upper abdomen. Ultrasonography suggested highly viscous contents, and magnetic resonance imaging showed no findings of malignancy. Given that the tumor markedly bulged into the pouch of Douglas, a transvaginal approach was selected to achieve safe decompression prior to trocar placement. Through a posterior colpotomy, the cyst wall was exposed extraperitoneally, enabling controlled extracorporeal incision and drainage of 9,782 mL of highly viscous fluid facilitated by manual abdominal compression. Tumor decompression enabled safe trocar insertion, pneumoperitoneum establishment, and laparoscopic adhesiolysis of dense adhesions to the abdominal wall and omentum. The adnexa were subsequently ligated and removed transvaginally without macroscopically evident intraperitoneal spillage of the tumor contents. Histopathology examination confirmed mucinous cystadenoma arising in a mature cystic teratoma. The patient experienced an uneventful recovery and was discharged on postoperative day 3. This case highlights that transvaginal drainage via posterior colpotomy is an effective strategy for giant ovarian cystic tumors with highly viscous contents, particularly when the tumor protrudes into the pouch of Douglas. Tailoring the surgical approach to the tumor’s anatomical characteristics can facilitate safe, minimally invasive management even in cases traditionally managed by laparotomy.

## Introduction

Laparoscopic surgery for ovarian tumors has been shown to significantly reduce postoperative complications and pain, as well as shorten hospital stays, compared with laparotomy [1]. In recent years, laparoscopic surgery has become the standard treatment for benign ovarian tumors. However, in cases of giant cystic ovarian tumors that completely occupy the abdominal cavity, trocar insertion and the establishment of an adequate operative field are often difficult, thereby increasing the risk of intraoperative capsule rupture and intraperitoneal spillage of tumor contents. Therefore, even when an ovarian tumor is considered benign, laparotomy is frequently selected.

Laparotomy requires a vertical midline incision proportional to tumor size, often extending from the pubic symphysis to the xiphoid process, resulting in considerable surgical invasiveness. Several laparoscopic techniques for giant ovarian cystic tumors have been reported, in which the tumor contents are aspirated laparoscopically to reduce tumor size prior to resection [2,3]. However, in mucinous tumors with highly viscous contents, aspiration with a suction device is often not effective.

Here, we report a case of a giant ovarian mucinous tumor with highly viscous contents that was successfully treated by transvaginal drainage of the tumor contents followed by laparoscopic resection. Reports describing minimally invasive management of giant ovarian mucinous tumors with highly viscous contents remain limited, particularly when the tumor occupies the entire abdominal cavity and precludes conventional laparoscopic decompression. This case highlights a practical surgical strategy for achieving controlled extracorporeal drainage and avoiding intraperitoneal spillage while preserving the benefits of minimally invasive surgery.

This case report was prepared in accordance with the CARE guidelines (Appendices).

## Case presentation

A 72-year-old gravida 2, para 2 woman presented with a six-month history of persistent abdominal distension. She had undergone a laparotomy for a mature cystic teratoma at the age of 22 years and had experienced menopause at 45 years of age. On physical examination, a large intra-abdominal mass extending from the pelvis to the xiphoid process was palpable. Vaginal examination revealed a mass bulging into the posterior fornix. Transabdominal ultrasonography revealed a multilocular cystic mass in the abdominal cavity. The mass contained hyperechoic punctate and linear echoes, suggesting highly viscous contents (Figure 1).

**Figure 1:**
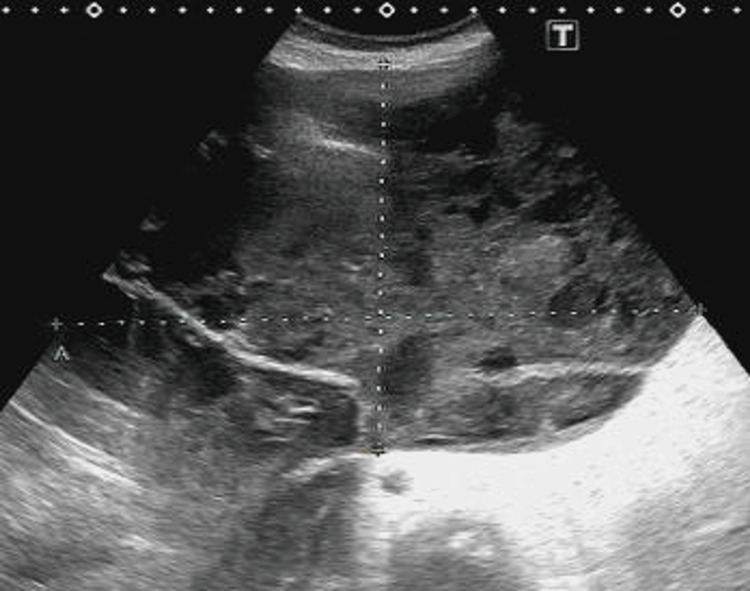
Transabdominal ultrasonography A multilocular cystic tumor occupying the abdominal cavity is shown. Hyperechoic punctate and linear echoes are visible within the cysts, suggesting highly viscous fluid components.

Magnetic resonance imaging (MRI) showed a 37.7 × 32.0 × 15.5 cm multilocular tumor bulging into the pouch of Douglas, with no septal thickening or mural nodules. The contents were hypointense on T1-weighted images and hyperintense on T2-weighted images, with no solid components (Figure 2).

**Figure 2:**
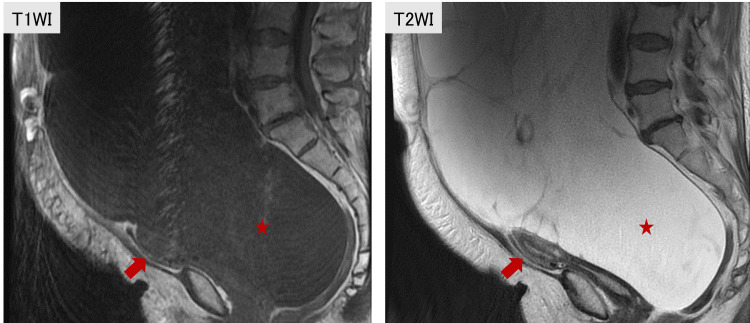
MRI imaging Left: sagittal T1-weighted image. Right: sagittal T2-weighted image. The tumor measures 37.7 × 32.0 × 15.5 cm and is bulging into the pouch of Douglas (star: tumor, arrow: uterus). The cyst contents demonstrate low signal intensity on T1‑weighted images and high signal intensity on T2‑weighted images. No solid components are observed.

Computed tomography (CT) demonstrated high-density areas within the tumor, suggestive of calcification (Figure 3).

**Figure 3:**
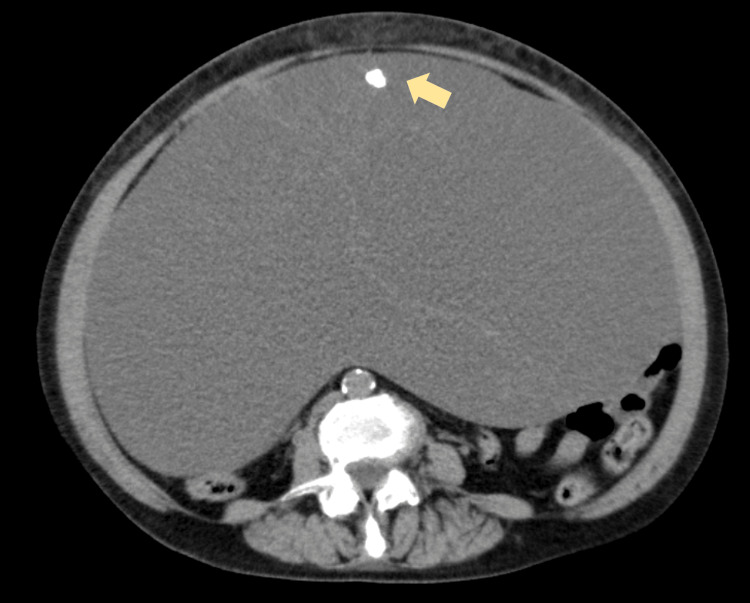
CT imaging CT reveals internal calcifications within the tumor (arrow).

The mass occupied nearly the entire abdominal cavity, extending from the pouch of Douglas to the upper abdomen. Serum tumor marker levels were elevated, including carcinoembryonic antigen, cancer antigen (CA) 19-9, CA125, and CA72-4 (Table 1).

**Table 1:** Preoperative serum tumor marker levels Preoperative serum tumor marker levels and their corresponding reference ranges.

Tumor Marker	Patient Value	Unit	Reference Range
Carcinoembryonic antigen	133.2	ng/mL	≤ 5.0 ng/mL
CA 19-9	43	U/mL	≤ 37 U/mL
CA125	46	U/mL	≤ 35 U/mL
CA72-4	94.9	U/mL	≤ 6.9 U/mL

A preoperative diagnosis was mature cystic teratoma with predominant mucinous components. Because there were no findings suggestive of malignancy and the patient desired minimally invasive surgery, laparoscopic surgery was planned.

We considered that laparoscopic surgery could be performed without intraperitoneal spillage of the cyst contents by taking advantage of the tumor bulging into the pouch of Douglas.

Surgical findings

Under general anesthesia, the patient was placed in the lithotomy position. After visualizing the posterior vaginal wall with a speculum, a transverse incision was made just below the posterior vaginal fornix to open the pouch of Douglas. The tumor bulged into the vagina, and with manual abdominal compression, it descended further toward the vaginal introitus. The tumor capsule was grasped with forceps and pulled out through the vagina, after which a small incision was made in the capsule extracorporeally. The tumor contents were highly viscous and difficult to aspirate; therefore, continuous manual abdominal compression was applied, and 9,782 mL of tumor fluid was drained transvaginally (Figure 4 A-D and Figure 5).

**Figure 4:**
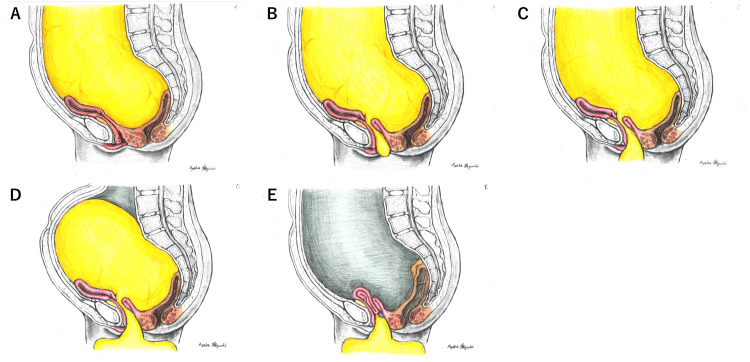
Transvaginal drainage of the ovarian tumor (A) The tumor is bulging into the pouch of Douglas. (B) A transverse incision is made in the posterior vaginal wall just below the posterior fornix, causing the tumor to protrude into the vaginal canal. (C) The tumor capsule is grasped and exteriorized, allowing extracorporeal incision of the capsule. (D) Transvaginal drainage of the cyst contents is performed, resulting in tumor reduction. (E) Transvaginal traction of the tumor allows ligation and division of the utero‑ovarian ligament and the infundibulopelvic ligament. Courtesy of Hidenori Oguchi. This schematic illustration was hand-drawn by the author and scanned for digital submission. No generative AI tools were used in the preparation of this figure.

**Figure 5:**
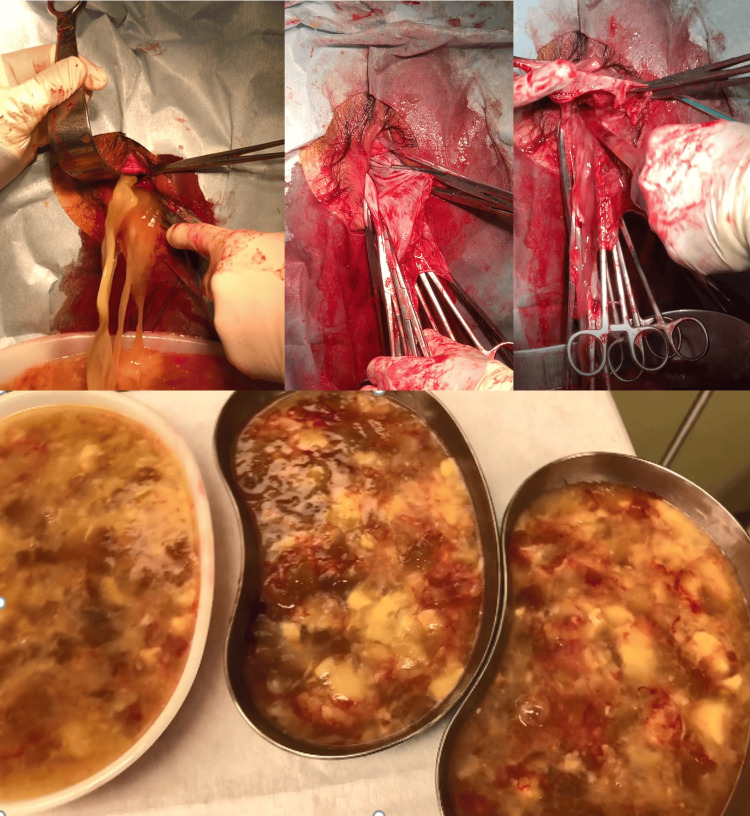
Transvaginal drainage of the mucinous fluid The tumor capsule is grasped with forceps and pulled outside the vagina. Because of the high viscosity of the cyst contents, aspiration is difficult. With manual abdominal compression, a total of 9,782 mL of viscous mucinous fluid is drained transvaginally.

After drainage, the abdominal distension was markedly reduced (Figure 6).

**Figure 6:**
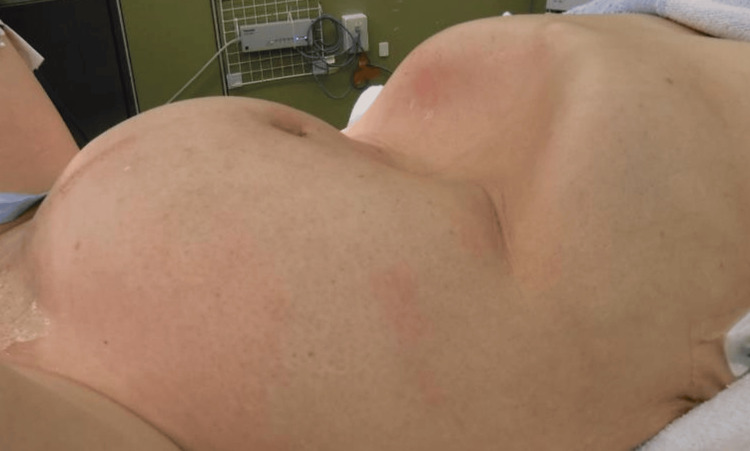
Abdominal appearance after drainage Reduction of the tumor size is evident on external abdominal inspection following drainage.

Regarding the prior laparotomy, adhesions between the tumor and intra-abdominal organs were anticipated; thus, adhesiolysis was planned laparoscopically. A uterine manipulator was inserted, and a 12-mm trocar was placed at the umbilicus using the open technique. The pneumoperitoneum pressure was set at 10 mmHg, followed by the insertion of 5‑mm trocars in the left lower quadrant and the lower midline, and a 12‑mm trocar in the right lower quadrant, resulting in a modified diamond trocar configuration (Figure 7A).

In laparoscopic observation, a right adnexal tumor reduced in size, remaining within the pelvic cavity (Figure 7B). The tumor was extensively adherent to the anterior abdominal wall and the omentum, and adhesiolysis was performed using monopolar energy (Figure 7C). No intraperitoneal spillage of tumor contents was observed. Because the left adnexa had been resected, only the right adnexa remained. The appendix showed no enlargement or other gross abnormalities, and no peritoneal dissemination or other findings suggestive of malignancy were observed. An additional 2,025 mL of tumor fluid was drained transvaginally under laparoscopic guidance (Figure 7D). Although it was difficult to identify the utero-ovarian ligament and infundibulopelvic ligament laparoscopically because of the tumor size, transvaginal traction of the adnexa allowed these structures to be identified directly (Figure 4E). The ligaments were ligated and divided transvaginally, and the right adnexa was removed and extracted transvaginally (Figure 7E).

**Figure 7:**
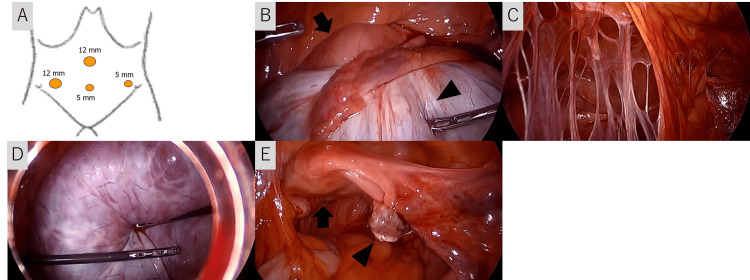
Surgical findings (A) Trocar placement (modified diamond configuration). (B) Intra‑abdominal appearance after initial drainage (arrow: uterus; arrowhead: right ovarian tumor). (C) Dense adhesions surrounding the tumor. (D) Additional transvaginal drainage of the tumor under laparoscopic guidance. (E) Intra‑abdominal appearance after right oophorectomy (arrow: uterus; arrowhead: stump of the suspensory ligament).

Throughout this process, the tumor remained under continuous transvaginal traction and was not returned to the peritoneal cavity. The specimen was subsequently removed directly through the vaginal incision under direct visualization without the use of a retrieval bag.

There was no apparent leakage of tumor contents into the peritoneal or vaginal cavity. The operative time was 4 hours 51 minutes, and blood loss was minimal. Total drained tumor fluid volume was 11,807 mL, and the right adnexa weight was 589 g. The tumor was a multilocular cystic mass with smooth internal and external surfaces (Figure 8).

**Figure 8:**
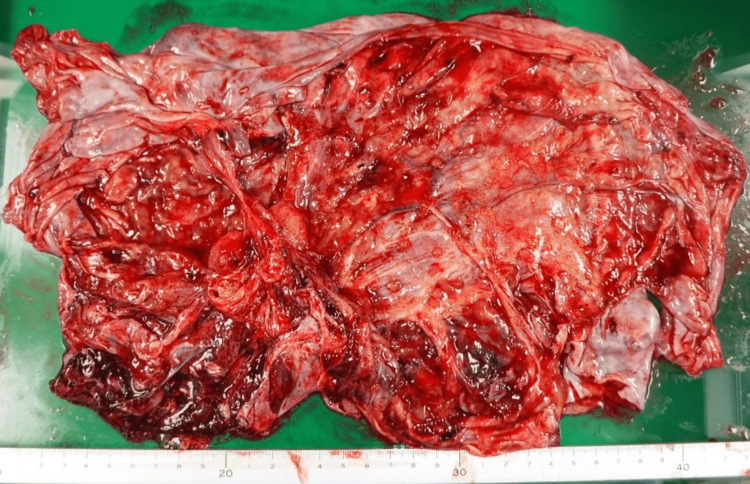
Macroscopic appearance of the right ovarian tumor A multilocular cystic mass with smooth internal and external surfaces.

Histopathology examination showed that most of the cyst wall was lined by a single layer of columnar epithelial cells with mucin production. Foci of squamous epithelium with keratinization and bone formation were also observed. The final diagnosis was mucinous cystadenoma arising in a mature cystic teratoma of the right ovary (Figure 9).

**Figure 9:**
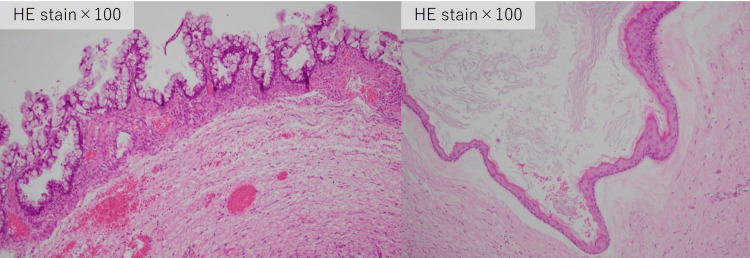
Histological findings of the right ovarian tumor (hematoxylin and eosin staining, ×100) Most of the cyst wall is lined by a single layer of mucin‑producing columnar epithelial cells. Foci of keratinizing squamous epithelium and areas of bone formation are also present.

The postoperative course was uneventful, and the patient was discharged on postoperative day 3. The tumor markers normalized within three months (Table 2).

**Table 2:** Serum tumor marker levels at three months after surgery All tumor marker levels normalized within three months after surgery.

Tumor Marker	Patient Value	Unit	Reference Range
Carcinoembryonic antigen	2.1	ng/mL	≤5.0 ng/mL
CA 19-9	4	U/mL	≤37 U/mL
CA125	10	U/mL	≤35 U/mL
CA72-4	2.8	U/mL	≤6.9 U/mL

The tumor markers normalized within three months. No postoperative complications occurred, and follow-up was completed at three months. The surgical video is shown in Video 1. 

**Video 1:** Transvaginal drainage of tumor contents

.

## Discussion

Although there is no strict definition of a “giant ovarian tumor,” many reports define it as a tumor measuring ≥10-15 cm in diameter or extending above the umbilicus [4,5].

Laparoscopic surgery for benign ovarian tumors offers several advantages over laparotomy, including reduced postoperative complications, pain, hospital stay, and cost [1]. However, when the tumor is giant, trocar placement and securing an adequate operative field become challenging, and the risk of capsule rupture and intraperitoneal spillage increases. In multilocular mucinous tumors with highly viscous contents, aspiration with a suction device is often ineffective, making laparoscopic management challenging. Consequently, laparotomy is often chosen even for tumors presumed benign [6].

Laparotomy for giant ovarian tumors has been associated with postoperative complications such as abscess formation, tissue necrosis resulting from redundant skin and muscle, and the need for abdominal wall reconstruction [7]. For cosmetic and morbidity reasons, minimally invasive approaches have been increasingly reported, but reports on giant ovarian mucinous tumors with highly viscous contents remain limited.

When performing laparoscopic surgery for ovarian tumors, appropriate patient selection is crucial. Preoperative ultrasonography and MRI should be used to exclude the possibility of malignancy as much as possible. In the present case, the patient exhibited elevated serum tumor marker levels, raising concern for borderline or malignant disease. On the other hand, MRI demonstrated no solid components, papillary projections, mural nodules, or septal thickening, and the imaging findings were more consistent with a benign mucinous lesion. The calculated Risk of Malignancy Index was 138, which was below the commonly used threshold of 200 for high-risk ovarian malignancy and supported the preoperative assessment of a low risk of malignancy [8]. This case illustrates the importance of integrating tumor marker results with detailed imaging findings when determining the surgical approach.

Nevertheless, the incidence of unexpected ovarian malignancy after laparoscopic surgery for presumed benign adnexal masses has been reported to be 0.04-2.08% [9-11], and malignancy cannot be completely ruled out. In addition, intraoperative capsule rupture of ovarian mucinous tumors has been suggested as a potential cause of pseudomyxoma peritonei [12]. Therefore, regardless of preoperative diagnosis, it is essential to remove the tumor without intraperitoneal spillage. In conventional laparoscopic surgery for adnexal masses, specimen retrieval bags are frequently used to reduce the risk of tumor content spillage during extraction. In the present case, however, the tumor remained under continuous transvaginal traction after decompression and was not returned to the peritoneal cavity. The infundibulopelvic ligament and utero-ovarian ligament were divided transvaginally while the specimen remained exteriorized. Consequently, direct transvaginal extraction was possible without the use of a retrieval bag, and no apparent intraperitoneal spillage was observed.

Various laparoscopic approaches for giant ovarian cystic tumors have been reported, and the surgical technique should be selected according to the consistency of the tumor contents and the morphology of the tumor, and pre-operative assumption is essential. Ultrasonography is useful for characterizing tumor contents; in mucinous tumors, high‑molecular‑weight glycoproteins increase the mucin concentration, leading to higher echogenicity [3]. In the present case, hyperechoic lines and dots suggested highly viscous mucin.

When the tumor does not occupy the entire abdominal cavity, an open technique can be used to place the camera port, with which pneumoperitoneum can be established and the cyst contents can be drained under laparoscopic visualization [2]. In contrast, when the tumor fills the abdominal cavity, drainage and tumor reduction must be performed prior to trocar insertion. Previous reports have described ultrasound‑guided puncture and drainage to reduce tumor size before initiating laparoscopy [13,14]. Although this method avoids additional abdominal incisions, it is difficult when a sufficiently large cyst is not accessible for safe puncture or when the cyst contents are highly viscous.

In the present case, the multilocular tumor occupied the abdominal cavity, and preoperative imaging suggested highly viscous contents. Because the tumor bulged into the pouch of Douglas, we selected transvaginal drainage via posterior colpotomy before trocar insertion. This approach avoids additional abdominal incisions and enables drainage under direct vision, thereby reducing the risks associated with blind puncture.

By creating a posterior colpotomy and applying manual abdominal compression, the tumor could be guided extracorporeally during drainage, preventing intraperitoneal spillage. Traction on the cyst wall under direct visualization allowed extracorporeal incision of the tumor wall and internal septa, enabling complete extracorporeal delivery of the tumor. Even in cases with highly viscous contents, such as ours, adequate drainage can be achieved by combining transvaginal access with manual abdominal compression. Although the patient had a history of laparotomy and dense adhesions between the tumor and intra-abdominal organs, transvaginal drainage and tumor reduction allowed safe laparoscopic visualization and successful adhesiolysis. Furthermore, transvaginal traction enabled direct visualization, ligation, and division of the utero‑ovarian and infundibulopelvic ligaments.

Transvaginal drainage is particularly suitable when the tumor bulges into the pouch of Douglas. At our institution, including the present case, three patients with giant ovarian cystic tumors extending to the xiphoid process have been successfully treated using this technique without intraperitoneal spillage.

## Conclusions

This case demonstrates that laparoscopic management of giant ovarian mucinous tumors with highly viscous contents is feasible when the surgical approach is carefully tailored to the tumor’s anatomical characteristics and internal consistency. In the present patient, the tumor occupied nearly the entire abdominal cavity, making conventional trocar placement and laparoscopic decompression technically challenging. Because the tumor protruded into the pouch of Douglas, transvaginal drainage through a posterior colpotomy enabled safe extracorporeal decompression of more than 11 liters of mucinous fluid without macroscopically evident intraperitoneal spillage. This reduction in tumor volume allowed adequate laparoscopic visualization, safe adhesiolysis, and successful adnexectomy through a minimally invasive approach.

This technique offers several practical advantages. It avoids an additional abdominal incision for decompression, facilitates management of highly viscous contents that are difficult to aspirate through conventional suction devices, and provides transvaginal traction that improves identification of key pelvic structures. In selected patients with giant benign-appearing ovarian cystic tumors that extend into the pouch of Douglas, this strategy may represent a useful minimally invasive surgical option while minimizing the risk of intraperitoneal rupture and spillage and preserving the benefits of minimally invasive surgery. Careful preoperative imaging assessment and appropriate patient selection remain essential for the safe application of this approach.

## Appendices

CAse REports (CARE) guidelines

This case report was prepared in accordance with the CARE guidelines [15].

**Table 3:** CARE guideline checklist Checklist of items recommended by the CARE (CAse REports) guidelines and their corresponding locations within the manuscript.

Topic	Item	Checklist Item Description	Location in Manuscript
Title	1	The diagnosis or intervention of primary focus followed by the words "case report"	Title
Keywords	2	2 to 5 keywords that identify diagnoses or interventions in this case report	Keywords
Abstract	3a	Introduction: What is unique about this case and what does it add to the scientific literature?	Abstract
Abstract	3b	Main symptoms and/or important clinical findings	Abstract
Abstract	3c	The main diagnoses, therapeutic interventions, and outcomes	Abstract
Abstract	3d	Conclusion: What is the main take-away lesson from this case?	Abstract
Introduction	4	One or two paragraphs summarizing why this case is unique	Introduction
Patient Information	5a	De-identified patient-specific information	Case Presentation
Patient Information	5b	Primary concerns and symptoms of the patient	Case Presentation
Patient Information	5c	Medical, family, and psychosocial history including relevant genetic information	Case Presentation
Patient Information	5d	Relevant past interventions with outcomes	Case Presentation
Clinical Findings	6	Significant physical examination findings	Case Presentation
Timeline	7	Historical and current information organized as a timeline	Not included
Diagnostic Assessment	8a	Diagnostic testing	Case Presentation
Diagnostic Assessment	8b	Diagnostic challenges	Not applicable
Diagnostic Assessment	8c	Diagnosis including differential diagnoses considered	Case Presentation
Diagnostic Assessment	8d	Prognosis where applicable	Not applicable
Therapeutic Intervention	9a	Types of therapeutic intervention	Surgical Findings
Therapeutic Intervention	9b	Administration of therapeutic intervention	Surgical Findings
Therapeutic Intervention	9c	Changes in therapeutic intervention with rationale	Not applicable
Follow-up and Outcomes	10a	Clinician- and patient-assessed outcomes	Case Presentation
Follow-up and Outcomes	10b	Important follow-up diagnostic and test results	Case Presentation
Follow-up and Outcomes	10c	Intervention adherence and tolerability	Case Presentation
Follow-up and Outcomes	10d	Adverse and unanticipated events	Case Presentation
Discussion	11a	Scientific discussion of strengths and limitations	Discussion
Discussion	11b	Discussion of relevant medical literature	Discussion
Discussion	11c	Scientific rationale for conclusions	Discussion
Discussion	11d	Primary take-away lessons	Conclusion
Patient Perspective	12	Patient perspective on the treatment received	Not included
Informed Consent	13	Patient informed consent obtained	Consent
